# Matrix Metalloproteinase (MMP)-9 Levels in Children on Hemodialysis: Association with MMP-9 C^-1562^T Gene Polymorphism and Vitamin D Levels

**Published:** 2016-09

**Authors:** Ashraf Galal, Fatina I. Fadel, Enas Mokhtar, Manal F. Elshamaa, Eman A. Elghoroury, Solaf Kamel, Gamila S. M. Elsaeed, Eman H. Thabet

**Affiliations:** 1 Department of Pediatric, National Research Centre, Cairo, Egypt;; 2 Department of Pediatric, Cairo University, Cairo, Egypt;; 3 Department of Clinical Pathology, National Research Centre, Cairo, Egypt;; 4 Department of Medical Biochemistry, National Research Centre, Cairo, Egypt

**Keywords:** ESRD, Vitamin D, MMP9, MMP9gene polymorphism, Hemodialysis, Children

## Abstract

Background and objectives: Data concerning the concentration of matrix metalloproteinase-9 (MMP-9) and its functional polymorphisms in chronic kidney diseases (CKD) are conflicting. The present study aimed to evaluate the levels of MMP-9in children with end stage renal diseases (ESRD) on hemodialysis (HD) and to explore its association with MMP-9 polymorphism and vitamin D levels as an important risk factors for cardiovascular diseases (CVD). Methods: We studied 55 children with ESRD on hemodialysis and 18 healthy children served as controls. MMP-9 and vitamin D levels were measured by ELISA in serum of all patients and controls. Genotypes for MMP-9 polymorphism(C^-1562^T) were determined by RFLP for only 28 of the patients and all the controls. Results: There were insignificantly reduced MMP-9levels of patients vs. controls, however, there was significant increase in MMP-9 levels associated with CC genotypes for(C^-1562^T) polymorphism compared with CT genotype (*p*=0.01). We found that at MMP-9 base position-1562, the frequencies of the genotypes CC and CT in Children on HD were 71.4% and 28.6% respectively while all our controls were of the CC genotype. The alleles frequencies of C and T in patients were 85.7% and 14.29% as compared to 100% and 0%, respectively in the controls. Significant decrease in vitamin D was observed in children on HD versus that in controls (*p*=0.008). Serum MMP9 levels and age were variables that were independently associated with CVD. Conclusions: MMP9 genetic polymorphism (C^-1562^T) affects MMP9alterations in ESRD children on HD and vitamin D deficiency is common in our HD pediatric patients who require attention in accordance with current practice guidelines. They probably require supplementation with higher doses of cholecalciferol.

## INTRODUCTION

Patients with chronic kidney disease (CKD) and on hemodialysis (HD) treatment are at higher dramatic cardiovascular risk than the general population (1). Even mild kidney dysfunction should be considered a medical condition predisposing to increased cardiovascular problem (2). Long term HD treatment, might aggravate atherosclerotic cardiovascular disease (CVD) in patients and according to increase mortality and morbidity of them (3). It is well known that extracellular matrix (ECM) turnover plays a critical role in the processing of CKD, and remodeling of ECM is an important physiologic feature of normal growth and development. Many diseases including CKD have been associated with an imbalance between ECM synthesis and degradation, which may result in accumulation of ECM molecules (4).

Matrix metalloproteinase (MMPs) are structurally related, zinc dependent proteins that disrupt the ECM and other non-ECM related substances (5). They are controlled at transcriptional and post translational levels, and their action will be likewise reliant on endogenous inhibitors, the tissue inhibitors of MMPs (TIMPs) (6). Mounting proof demonstrates that imbalanced MMP activity contributes to atherosclerotic diseased vessels in patients with renal failure (7), especially MMP-9 which is correlated with atherosclerosis in non-dialytic chronic kidney disease (8).

MMP-9 activity is highly dependent on its expression level (6), and functional genetic polymorphisms in the MMP-9 gene might influence MMP-9 concentrations (9), conceivably modifying the susceptibility to cardiovascular diseases (CVD) (10).

The MMP-9 gene has three major polymorphisms in the promoter, coding and untranslated regions (11). Of these, the C^-1562^T (rs 3918242) polymorphism in the promoter region is of special interest.

Vitamin D is obtained from both the diet and upon exposure to ultraviolet light (12).

Precursors of vitamin D2 or D3 are hydroxylated in the liver to form 25-hydroxyvitamin D [25(OH) D]. A second hydroxylation occurs primarily in the kidney, leading to formation of 1, 25-dihydroxyvitamin D [1, 25(OH) 2D] (calcitriol). Calcitriol regulates intestinal absorption of calcium, bone resorption, and renal excretion of calcium and phosphate. Significant alterations in renal function, such as those found in CKD, result in decreased activity of renal 1-alpha-hydroxylase (13), leading to decreased production of calcitriol. This in turn leads to decreased intestinal absorption of calcium and reduced renal phosphate excretion, resulting in hypocalcemia and hyperphosphatemia. Hypocalcemia reduces the activity of the calcium-sensing receptor in the parathyroid gland and stimulates the secretion of PTH (14). PTH, in response to both low serum calcium and elevated serum phosphate levels, increases the tubular reabsorption of calcium and the tubular secretion of phosphate, and stimulates renal enzyme 1α hydroxylase to produce 1,25(OH)2D. However, as noted above, patients with CKD are not able to produce adequate amounts of 1, 25(OH) 2D. These patients may also have poor baseline nutritional intake related to uremia-induced reduction of appetite, as well as dietary restriction of specific nutrients, such as phosphorus, leading to inadequate substrate for conversion to calcitriol. Beyond its effects on bone, vitamin D deficiency has been associated with multiple other deleterious effects, including hypertension, cardiovascular morbidity, inflammation, and cancer risk (15, 16).

Observational studies suggest an inverse association between circulating 25-hydroxyvitamin D [25(OH) D] concentration and serum inflammatory biomarkers. Low 25(OH) D concentrations are associated with elevated tumor necrosis factor-alpha (TNF-α) concentrations (17), and randomized controlled studies among critically ill patients and those with congestive heart failure report that vitamin D supplementation leads to significant reductions in interleukin 6 (IL-6) and C-reactive protein (CRP) concentrations (18), and resulted in lower tumor necrosis factor-alpha (TNF-α) and increased anti-inflammatory IL-10 concentrations (19). However, few studies have addressed the association between 25(OH) D concentration and increased inflammation on biomarkers of vascular remodeling. MMP’s) are important in extracellular matrix remodeling , are major effector molecules of inflammatory cells, and play a key role in many vascular diseases, including hypertension and aneurysm formation (20, 21). Some *in vitro* studies indicate that vitamin D down-regulates MMP-9 production by TNF-α (22) and suppresses production of MMP-7 and MMP-9 (23-25). Moreover, MMP-2 and MMP-9 activation are associated with collagen deposition and cardiac fibrosis (26). Previous studies have also suggested that vitamin D deficiency in CKD patients is associated with higher cardiovascular diseases and mortality (27, 28).

The data on MMP-9 concentrations and its functional polymorphism in CKD patients are scarce and contradictory and concerns only adults. This study was undertaken to evaluate the level of MMP-9 in children with end stage kidney diseases (ESRD) on HD and to explore its association with functional MMP-9 polymorphism(C^-1562^T) and vitamin D levels in these children.

## MATERIALS AND METHODS

55 children with ESRD undergo hemodialysis at the hemodialysis unit of the Center of Pediatric Nephrology and Transplantation (CPNT), children’s hospital, Cairo University were included in the study. Etiology of ESRD was as follows: hereditary nephropathies in 17 patients, obstructive uropathies in 13, glomerulopathy in 9, renal hypoplasia or dysplasia in 5, metabolic causes in 3 and was unknown in 8 cases. All patients were dialyzed using a polysulfone dialyzer, with bicarbonate dialysate, using a blood flow rate of 80-150 ml/min and are dialyzed 3 times per week using polysulfone membranes. The dialysate fluids were prepared from concentrated salt solutions and from bicarbonate powder in sealed containers. As recommended by the FDA, the water purification system combines a double softener with a double granular charcoal filter and a double reverse osmosis module in series. Inclusion criteria included children on regular HD treatment for not less than 4 months, using bicarbonate dialysate and free from apparent acute illness. Patients with severe illness or acute medical events were excluded.

Patients had taken routine medications in a dialysis unit for at least 3 months, such as phosphate-binding agents, calcium carbonate (35 patients (81.40%), 500 mg/tablet (Ca 0.2 g, 10 mEq) at a dose of 500-4000 mg/day, or calcium acetate (8 patients (18.60%), 500mg/tablet (Ca 0.2 g, 10 mEq) at a dose of 1500-2000 mg/day. Patients with parathormone (PTH) level greater than 100 pg/ml or hypocalcaemia received daily rocaltrol (38 patients (88.37%), at a dose of 0.25-2 µg/day. Antihypertensive medications taken by the patients were as follows: calcium channel antagonists (n=36), angiotensin converting enzyme inhibitors (n=24) and B-blockers (n=6). Medication compliance was checked by questionnaires and re-enforced at each dialysis unit. An EPO 750-9000 IU/week, subcutaneous injection was administered to maintain hematocrit between 28% and 31%. Eighteen healthy age- and gender-matched children were recruited from the pediatric clinic of the National Research Centre (NRC) to serve as controls. Written consent was obtained from the parents of each patient. The study was approved by ethical committees of both NRC in Egypt and CPNT, Children hospital, Cairo University. All patients were subjected to full history taking and thorough clinical examination.

### Diagnostic criteria of vascular disease

We studied the prevalence of vascular disease in children with CKD according to the following criteria (29).

Cardiac disease: the presence of primary dilated cardiomyopathy previously diagnosed clinically and by echocardiography.

Peripheral vascular or cerebral vascular diseases: Cerebral vascular disease was suspected on clinical grounds, that is, rapidly developing signs of focal disturbance of cerebral function such as hemiparesis and hemi-sensory impairment. The diagnosis was confirmed by computed tomography or magnetic resonance imaging. Brain hemorrhage and subarachnoid hemorrhage were excluded.

A patient was considered to have a vascular disease when at least one of these two defined vascular disease was present.

### Blood Sampling

A peripheral blood sample was obtained prior to the hemodialysis session from the venous part of the arteriovenous fistula using a specially selected disposable plastic syringe. The 3 ml blood was divided into 2 ml to be centrifuged and 1ml blood sample on EDTA for gene analysis. The 2 ml blood sample was transferred to a sterile plastic vaccutainer tube and then immediate centrifugation was done for 10 min at 5000 rpm at 4°C and the one on EDTA was stored at -20°C should be analysed within 6 mothns .If later analysis will be done the samples must be frozen at -70°C.

All biochemical parameters were measured for all patients using an automatic biochemistry analysis. Serum 25-hydroxyvitamin D level was measured by ELISA (DRG, GmbH, Germany) Patients were labeled vitamin D insufficient if the levels were between 20 and 30 ng/ml, and deficient if the levels were less than 20 ng/ml. We also subcategorized these patients to severe vitamin D deficiency if the level was less than 10 ng/ml.

Serum MMP9 was measured by ELISA (L140624541 lot no. Alamo laboratory, USA).

### Genotyping

MMP9 gene analysis for the(C^-1562^T) polymorphism was done for only 28 patients and 18 healthy controls because these were the available numbers of EDTA samples, also the SphI (Fermentas) enzyme used for digestion of PCR products digests only 10 samples and the budget of the research was not enough to analyze more cases.

### DNA extraction

One ml blood were used for DNA extraction using the QIA amp® DNA blood mini kit (Qiagen Sciences, Germantown, MD, USA) according to manufacturer’s instructions. DNA was stored frozen at −20°C until processed.

### PCR for detection of MMP-9C^-1562^T gene promoter polymorphism

DNA concentration was determined by Nano Drop 2000 c Spectrophotometer (Thermo Fisher) and diluted as100 ng/μl .Genomic DNA was amplified using polymerase chain reaction (PCR), amplification was carried out on a Veriti thermal cycler Applied Biosystems, USA, in a 25 μl reaction mixture containing 200ng genomic DNA, 12.5 μL master mix using Taq DNA Polymerase (Applied Biosystems), 5 p mol each primers 5′-GCC TGG CAC ATA GTA GGC CC-3′ (sense) and 5′-CTT CCT AGC CAG CCG GCA TC-3′ (antisense). Samples were initially denatured at 95°C for 3-min, followed by 35 cycles of 95°C for 1-min, 65°C for 45-s, 72°C for 45-s, and a final extension step of 72°C for 7-min. PCR products were analyzed by 1.5% agarose gel electrophoresis, stained with ethidium bromide and a fragment of 435 bp was visualized in ultraviolet transilluminator (Biometra).

### Restriction endonuclease digestion (RFLP)

PCR products were digested with SphI (Fermentas) in a volume of 20 μL containing Digestions were made in a thermal cycler plate at 37°C. Finally, the optimal digestion parameters selected were 1 μg DNA and 1 U enzyme during 2-h of incubation. Fragments were separated in agarose 1.5% stained with ethidium bromide. The C allele was not cut by SphI giving a fragment of 435 bp and the T allele was cut into fragments of 247 and 188 bp.

### Statistical Analysis

SPSS for Windows, version 15.0 computer program was used for statistical analysis. Data are represented as the mean ± SD and as frequency and percentages accordingly. A p value of less than 0.05 was considered statistically significant. The t--test was used to compare between 2 independent means. Chi-square and Fisher’s exact tests were used to compare between independent proportions. Pearson correlation coefficient “r” was used to measure the linear relationship between different continuous variables. Multiple regression analysis was performed to assess the influence of risk factors on CVD and binary logistic regression was performed to assess the influence of MMP-9 alleles on CVD. Power analysis was used to calculate the minimum sample size required to accept the outcome of a statistical test with a particular level of confidence. A sample size of 20 will give us approximately 80% power (alpha = 0.05, two-tail) to reject the null hypothesis of zero correlation. We used power calculations performed by the Power and Precision program (Biostat) to determine the number of chromosomes required to detect a significant difference between the polymorphism frequency in the reference population and the expected frequency. Power commonly sets at 80%; however, at that level, a polymorphism would be missed 20% of the time. The Hardy- Weinberg equilibrium (HWE) assumption was assessed for case and control groups by comparing the observed numbers of different genotypes with those expected under HWE for the estimated allele frequency and comparing the Person goodness-of-fit statistic with a 2 distribution with 1 degree of freedom.

## RESULTS

Detailed characteristics of patients and controls are shown in Table 1. Mean age, sex, body mass index and vascular access are presented in the table with no statistically significant difference with controls. We noted that 78.19% of the patients were hypertensive while 21.81% of the patients and all the controls were normotensive (*P*=0.01). We also noted a significant increase in creatinine (*P*=0.0001), cholesterol (*P*=0.0027), triglycerides (*P*=0.0001), and significantly decreased HDL-cholesterol levels (*P*=0.0001) in patients vs. controls. MMP-9 levels were insignificantly reduced in patients as compared to controls. SD was greater than the mean value of Alkaline Phosphatase for the patients group and the TPC group in Table 1 and Table 2 respectively, due to variability of the results (range of all patients. 70-3428(U/L) which is a common finding in the value of Alkaline Phosphatase in serum. Only 13.3% of patients were vitamin D deficient and 20% were vitamin D insufficient. The remaining 66.7% were vitamin D sufficient, all of these patients were on usual calcium supplements and 38 patients were on calcitriol. When comparing our patients to healthy controls, serum vitamin D levels were significantly lower in patients than in controls (*P*=0.0085).

**Table 1:** Demographic and clinical characteristics of children with ESRD on HD and healthy controls

	HD Children (n=55)	Controls (n=18)	*P* value

Age (years)	9.9 ± 3.88	10 ± 8.8	0.074
Gender (M/F)	25 (45.45%)/30 (54.55%)	11 (61.11%) /7 (38.89%)	
BM I(Kg/m2)	17.1 ± 4.8	20.6 ± 1.44	0.162
KT/V	3.84 ± 1.13		
Activated vitamin D treatment	55 (100%)		
Vascular access type			
AVF	44 (80%)		
TPC	11 (20%)		
Blood pressure			
Hypertensive (%)	43 (78.19%)		
Normotensive (%)	12 (21.81%)	18 (100%)	0.01
Peripheral Vascular (%)	9 (16.37%)		
Any CV disease (%)	11 (20%)		
Creatinine (mg/dl)	6.30 ± 1.45	0.73 ± 0.33	0.0001*
Hemoglobin (g/dl)	10.29 ± 1.93	14.23 ± 1.50	0.933
Albumin (g/dl)	3.542 ± 0.573	4.92 ± 0.39	0.329
Calcium (mg/dl)	8.777 ± 1.45	10.91 ± 0.40	0.316
Phosphorous (mg/dl)	4.388 ± 1.578	3.90 ± 0.58	0.561
Alkaline Phosphatase (U/L)	559.42 ± 580.14	134.0 + 113.0	0.612
Cholesterol (mg/dl)	192.04 + 50.37	161.31 ± 18.75	0.0027*
Triglycerides (mg/dl)	146 ± 50.37	65.98 ± 17.35	0.0001*
HDL-cholesterol (mg/dl)	27.33 ± 9.87	40.55 ± 7.83	0.0001*
Vitamin D (ng/ml)	37.6 ± 18.38	51.2 ± 20.24	0.0085*
MMP9 (pg/ml)	157.91 ± 48.43	172.17 ± 33.01	0.48

Results are expressed as number (%) or mean ± standard deviation. ESRD, end stage renal disease; HD, hemodialysis; BMI,mbody mass index; KT/V, dialysis adequacy; AVF, arteriovenous fistula; TPC, tunneled permanent catheter; MMP9,mmatrix metalloproteinase 9;

* *P*<0.05 was considered significant.

**Table 2:** Characteristics of hemodialysis children by vascular access type

	Tunneled permanent catheter (TPC) (n=11)	Arteriovenous Fistula (AVF) (n=44)	*P* value

Age(years)	8.80 ± 4.63	10.09 ± 3.59	0.30
Gender(M/F)	5 (45.45%)/6 (54.55%)	20 (45.45)/24 (54.55%)	0.98
Blood pressure			
Hypertensive (%)	5 (45.45%)	38 (86.36%)	0.43
Normotensive (%)	6 (54.55%)	6 (13.64%)	
Peripheral Vascular (%)	6 (54.55%)	3 (6.82%)	0.18
Any CV disease (%)	4 (36.36%)	7 (15.91%)	
Creatinine (mg/dl)	5.16 ± 1.61	6.14 ± 1.98	0.18
Hemoglobin (g/dl)	10.31 ± 2.49	10.31 ± 1.69	0.99
Albumin (g/dl)	3.3867 ± 0.71	3.61 ± 0.49	0.23
Calcium (mg/dl)	8.51 ± 1.24	8.90 ± 1.57	0.40
Phosphorous (mg/dl)	4.17 ± 1.67	4.51 ± 1.57	0.49
Alkaline phosphatase (U/L)	634.93 ± 828.29	525.44 ± 443.18	0.56
Cholesterol (mg/dl)	199.88 ± 60.88	188.20 ± 42.47	0.60
Triglycerides (mg/dl)	110.40 ± 45.80	158.30 ± 76.32	0.22
HDL-cholesterol(mg/dl)	33.33 ± 20.08	28.64 ± 12.09	0.52
Vitamin D (ng/ml)	33.00 ± 9.90	42.55 ± 1.04	0.49
MMP9 (pg/ml)	157.47 ± 61.92	152.50 ± 39.37	0.74

Results are expressed as number (%) or mean ± standard deviation. MMP9, matrix metalloproteinase 9, *P*<0.05 was considered significant.

The characteristics of the patients by vascular access type are summarized in Table 2, which showed no statistically significant differences between types of vascular access as regards to all measured parameters.

The distribution of genotypes and alleles of MMP-9 C^-1562^T gene polymorphism in patients and controls is displayed in Table 3. Gene analysis was done for only 28 patients and all the controls. Whilst the CC genotype was observed at frequency of 71.4% and 100% of patients and controls, respectively, the CT genotype was only observed in 28.6% of patients and none of the controls. The frequencies of C and T alleles were 85.71% inpatients vs.100%of controls and 14.29% of patients vs. none of the controls, respectively. Hardy-Weinberg equilibrium equations is balanced (equal 1) in each group. The difference in frequencies of both genotypes and alleles were not statistically significant (*P*=0.3 for both genotypes and alleles).

**Table 3:** Genotypes and alleles distribution of C^-1562^T gene polymorphism in MMP-9 gene in patients and controls

	HD children (n=28)	Control (n=18)	*P* value

Genotypes			
CC	20 (71.4%)	18 (100%)	0.30
CT	8 (28.6%)	0 (0%)	
Alleles			
C	48(85.71%)	36(100%)	0.34
T	8(14.29%)	0(0%)	

HD, hemodialysis, *P*<0.05 was considered significant.

As the patients were divided according to the genotypes for the C^-1562^T polymorphism, we had statistically significant increase in MMP-9 levels in CC genotypes patients compared to CT genotypes patients (*P*=0.01) as shown in Table 4.

**Table 4:** Comparison between CC and CT genotypes for the (C^-1562^T) MMP-9 gene polymorphism in children on HD

	CC (no =20)	CT (n=8)	*P* Value

Age (years)	9.69 ± 3.91	8.71 ± 3.29	0.547
BMI (Kg/m^2^)	16.20 ± 1.42	16.50 ± 2.10	0.576
KT/V	2.5 ± 1.63	1.86 ± 0.32	0.291
Hemoglobin (g/dl)	10.59 ± 1.87	10.3 ± 1.76	0.763
Albumin (g/dl)	3.7 ± 0.5	3.35 ± 0.39	0.089
Calcium (mg/dl)	8.79 ± 1.10	8.9 ± 1.98	0.838
Phosphorus (mg/dl)	4.47 ± 1.69	4.76 ± 1.49	0.678
Alkaline phosphatase (U/L)	540.89 ± 448.49	491.88 ± 328.20	0.783
MMP-9 (pg/ml)	165.15 ± 56.44	131.13 ± 23.87	0.012*
Vitamin D (ng/ml)	44.86 ± 20.23	36.25 ± 12.50	0.437

HD, hemodialysis; BMI, body mass index; KT/V, dialysis adequacy; MMP9, matrix metalloproteinase 9;

* *P*<0.05 was considered significant.

There was no significant difference between patients with CVD and those without in regards to serum MMP-9 levels (158.25 ± 56.38 pg/ml vs157.71 ± 44.15 pg/ml, *p*=0.97) or vitamin D (33.00 ± 13.71 ng/ml vs 39.90 ± 20.61 ng/ml, *p*=0.51) (Table 5).

**Table 5:** Comparison of clinical parameters in children with or without CVD

	CVD (+) (n=20)	CVD (-) (n=35)	*P* value

Gender (males/females)	10 (50%)/10 (50%)	16 (45.71%)/19 (54.29%)	0.82
Age (years)	10.50 ± 4.43	9.1429 ± 3.50094	0.25
Hypertensive (%)	20 (100%)	23 (65.71%)	0.15
Hemoglobin (g/dl)	10.76 ± 2.02	9.97 ± 1.83	0.16
Albumin (g/dl)	3.65 ± 0.49	3.47 ± 0.62	0.30
MMP-9 (pg/ml)	158.25 ± 56.38	157.71 ± 44.15	0.97
Vitamin D (ng/ml)	33.00 ± 13.71	39.90 ± 20.61	0.51

*P*<0.05 was considered significant.

The correlation between vitamin D and MMP-9 levels with different variables in HD patients are shown in Table (6).There is no significant correlation between MMP-9 levels and vitamin D levels (r=-0.15, *P*=0.59).

**Table 6:** Correlation between Vitamin D and MMP9 levels with different variables in HD children

	Vitamin D		MMP-9	
	r	*P* value	r	*P* value

Age (years)	0.39	0.24	-0.22	0.10
BMI (Kg/m^2^)	0.25	0.43	0.21	0.16
KT/V	-0.05	0.88	-0.04	0.76
Hemoglobin (g/dl)	-0.10	0.75	-0.15	0.32
Albumin (g/dl)	0.01	0.96	0.10	0.49
Calcium (mg/dl)	0.03	0.94	-0.07	0.65
Phosphorous (mg/dl)	0.25	0.41	0.29	0.05
Alkaline phosphatase (U/L)	-0.26	0.39	0.02	0.92
MMP9 (pg/ml)	-0.15	0.59	1	1
Vitamin D (ng/ml)	1	1	-0.15	0.59

HD, hemodialysis; BMI, body mass index; KT/V, dialysis adequacy; MMP9, matrix metalloproteinase 9; *P*<0.05 was considered significant.

Multiple linear regression analysis demonstrated that the risk factors for CVD in patients on HD were serum MMP9 (β=0.838, *P*=0.023) and age (β=0.658, *P*=0.017). (Table 7). Also vitamin D deficiency was insignificantly associated with CVD (β=-0.712, *P*=0.05).

**Table 7:** Risk factors affecting CVD in HD children based on multiple linear regression analysis

	β	*P* value

Age (years)	0.658	0.017*
Hypertensive (%)	0.675	0.084
MMP9 (pg/ml)	0.838	0.023*
Vitamin D (ng/ml)	-0.712	0.050
C allele	0.531	0.260

Based on multiple linear regression analysis and binary logistic regression for C allele. HD, hemodialysis; MMP9, matrix metalloproteinase 9;

* *P*<0.05 was considered significant.

## DISCUSSION

Patients with end stage renal disease (ESRD) bear an overwhelming risk of CVD (30). The vascular alterations present in ESRD are related to extracellular matrix remodeling and elastocalcinosis (31), and include imbalanced matrix metalloproteinase (MMP) activity (32). In the present study we investigated the correlation of MMP-9 serum concentration and MMP-9 gene polymorphism in children with ESRD on hemodialysis. To our knowledge, this is the first study to assess the relationship of MMP-9C^-1562^T gene polymorphism and its serum level in children with ESRD on HD. Our results showed reduced levels of circulating MMP-9 in HD patients compared to the controls but the difference was not statistically significant.

This finding is consistent with (33, 34). Furthermore, Ebihara *et al* (35) reported that chronic dialysis caused expression of the mRNA MMP-9 in peripheral blood monocytes without significant rise of serum MMP-9. However our results differ from preceding reports which described elevated or unaltered MMP-9 levels in HD patients. Pawlak *et al* (36) did not find difference in the MMP-9 levels between the hemodialysis patients and healthy controls; while in Musial and Zwolinska (37), MMP-9 was significantly elevated in all hemodialysis children versus controls. It will be probable that the inconsistence in described MMP-9 levels is related to the complex regulation of inflammation and MMPs; as well as differences in sample size, populations, anti-coagulation system, vascular access, type of membrane and causative diseases for ESRD. Moreover, such discrepancies may result from methodology or differences in the profile of examined groups, also matrix metalloproteinases are subject to different factors modifying their activity. The levels of MMPs depend on the presence of diabetic nephropathy, hypercholesterolemia, increased oxidative stress, ACE inhibitors, and hypertension (38-40).

Taken into consideration, the alterations in circulating MMP-9 levels in HD patients appear to be a result of the combination of many factors. We cannot find any significant association between MMP-9 levels and the other examined parameters in our patients.

In our study, we found that at MMP-9 base position-1562, the frequencies of the genotypes CC and CT in cases were 71.4% and 28.6% respectively, while 100% of our controls were of the CC genotype, and the allele frequencies of C and T in patients were 85.71% and 14.29% respectively as compared to 100% and 0% respectively, in our controls. Hardy-Weinberg equilibrium equations is balanced without evolution (equal 1) in each group because of not experiencing the following conditions; mutation, immigration /emigration, natural selection or sexual selection.

No statistically significant difference was detected in genotype or allele frequency between these groups. However, a statistically significant difference in serum levels of MMP-9 was observed among children with different genotypes, where MMP-9 levels among patients with CC genotypes were significantly higher than that of CT genotypes. This result suggests that this polymorphism may have effects in ESRD patients on HD at least in terms of circulating MMP-9 levels. Marson *et al* (41) reported that MMP-9 polymorphism do not significantly influence baseline MMP-9 levels in ESRD patients, while they reported a significant genetic contribution of MMP-9 polymorphism to hemodialysis induced alterations in MMP-9 levels in their study, HD increased MMP-9 levels significantly in subject with CC genotype but not in subject with CT or TT genotype for this polymorphism.

Some observations have linked a suboptimal 25(OH) D status to myopathy and adverse cardiovascular outcomes and also to the rate of progression of renal insufficiency in CKD (42-44). In the present study, we found a significant decrease in vitamin D level in HD children compared to the controls. Our findings are consistent with previous data (45, 46). Of note, all of our patients were on calcium carbonate or calcium acetate. Despite taking daily Rocaltrol, vitamin D levels were low in our patients.

Impaired metabolism of vitamin D is a common feature of CKD, however, insufficient intake of protein and calories as a result of decreased appetite or protein restriction may explain vitamin D deficiency in our patients. Another reason might be decreased exposure to sunlight due to restricted physical activity for a variety of reasons (47). We did not find any correlation between vitamin D and MMP-9 concentrations although an inverse correlation has been shown in the study of Wasse *et al* (48), who found that the vascular access type modifies the relation between MMP-9 and vitamin D concentrations. When they stratified AVF versus AVG (arteriovenous graft), the association between MMP-9 and vitamin D concentration no longer remained significant among AVF patients , whereas the relationship remained significant among patients using an AVG , suggesting that MMP-9 concentrations in AVF patients were too low for a relationship with vitamin D to be detected. This can be explained by the presence of the prosthetic material within an AVG which induce increased systemic inflammation. There is no patients in our study used an AVG and most of our patients were using AVF which can also explain why we found no statistically significant differences between different types of vascular access as regards to MMP-9and vitamin D levels.

Our findings suggest that vitamin D deficiency is common in our HD pediatric patients, which require attention in accordance with current practice guidelines. They probably require supplementation with higher doses of cholecalciferol. Further studies on supplementation are being recommended to define optimum dose schedule.

On correlating CVD to different risk markers by multiple linear regression analysis, we found that serum MMP9 levels and were the variable that were independently associated with CVD. Inflammation, lipid disturbances, endothelial damage, and autoimmunity play a pivotal role in atherogenesis (49). MMPs represent a family of endopeptidases with the proteolytic activity, targeting the components of extracellular matrix. Their activity can be regulated by several factors, such as oxidative stress (40). Such a variety of control pathways may be responsible for their ambiguous effects. In detail, MMP-9 (gelatinase B) has been described in in vitro investigations as a protector of the plaque stability in its early stages and the promoter of the increased vulnerability through hemorrhage in the advanced lesions (50, 51).

This study has some limitations that should be taken in consideration. The sample size is relatively small, which may limit the statistical power to find the difference between groups, thus future studies with larger sample size may contribute to elucidate the impact of this polymorphism on the risk of CVD in ESRD. Moreover, the assays used in the present study are based on antigen detection and may not reflect activity, particularly where a genotypic difference may affect activity.

Conclusion, our findings support the concept that MMP-9 genetic polymorphism affects MMP-9 changes in ESRD patients on HD. Where ESRD patients with CC genotypes for the C^-1562^T polymorphism are exposed to significant increase in MMP-9 levels than with CT genotypes, (whether these patients will benefit from the usage of MMP-9 inhibitors remains to be clarified). Serum MMP9 levels and age were variables that were independently associated with CVD. Our findings also suggest that vitamin D deficiency is common in our HD pediatric patients and they probably require supplementation with higher doses of cholecalciferol Further studies on supplementation are recommended to define optimum dose schedules.
